# Red Meat-Derived Nitroso Compounds, Lipid Peroxidation Products and Colorectal Cancer

**DOI:** 10.3390/foods8070252

**Published:** 2019-07-11

**Authors:** Pablo Steinberg

**Affiliations:** Max Rubner-Institut, Federal Research Institute of Nutrition and Food, Haid-und-Neu-Str. 9, 76131 Karlsruhe, Germany; pablo.steinberg@mri.bund.de; Tel.: +49-721-6625-201

**Keywords:** colon cancer, endogenously formed nitroso compounds, lipid peroxidation products, red meat

## Abstract

About 20 years ago, the research group of Sheila Anne Bingham in Cambridge, UK, showed for the first time that volunteers consuming large amounts of red meat excrete high amounts of nitroso compounds via feces. In the meantime, it has been demonstrated that heme leads to the enhanced formation of nitroso compounds in the gastrointestinal tract and that the main nitroso compounds formed in the gastrointestinal tract are *S*-nitrosothiols and the nitrosyl heme. Moreover, it has been postulated that these endogenously formed nitroso compounds may alkylate guanine at the *O*^6^-position, resulting in the formation of the promutagenic DNA lesions *O*^6^-methylguanine and *O*^6^-carboxymethylguanine, which, if not repaired (in time), could lead to gene mutations and, subsequently to the development of colorectal cancer. Alternatively, it has been postulated that heme iron could contribute to colorectal carcinogenesis by inducing lipid peroxidation. In the present review, the evidence supporting the above-mentioned hypotheses will be presented.

## 1. Introduction

In Western countries, malignant tumors of the colon and rectum are one of the most frequently observed cancer types (taken together, 10% of the total number of cancers) [1]. There were over 1.8 million new cases of colorectal cancer worldwide in 2018 [1] and about 70,000 new cases alone in Germany [2]. In 10% of the cases, the patients inherit genetic changes that strongly increase the risk of developing a tumor in the colon and/or rectum. In the rest of the cases, various different risk factors such as smoking, lack of physical activity, a low consumption of dietary fiber, a fat- and calorie-rich diet as well as the frequent consumption of high amounts of strongly heated meat have been postulated to increase the risk of developing colorectal cancer in humans. As early as in 1999, a scientific committee of the World Health Organization suggested that the consumption of red and in particular strongly heated meat correlates with an increased risk to develop a malignant tumor in the colon and rectum [3]. In the meantime, epidemiological studies in North America and Europe indeed showed that a positive correlation between the consumption of strongly heated meat and the development of colorectal tumors does in fact exist [4,5,6,7,8]. In this context, various different groups of compounds such as polycyclic aromatic hydrocarbons, heterocyclic aromatic amines and exogenously formed *N*-nitroso compounds, which are formed when meat is heated, have been suggested to play an important role in colorectal carcinogenesis [4,5,6]. In the last 20 years, evidence has been accumulating that a new group of compounds, so-called endogenously formed nitroso compounds, should also be viewed as potential human colorectal carcinogens. In the present review, the endogenous formation of nitroso compounds as well as the postulated mechanisms by which they could transform epithelial cells in the human colon and rectum will be described. Moreover, the potential role of red meat-derived lipid peroxidation products in colorectal cancer development will be discussed. A review of population-based studies linking the consumption of red meat, endogenously formed nitroso compounds and heme iron to colorectal cancer development is not included in this commentary.

## 2. The Formation of Nitroso Compounds in the Human Gastrointestinal Tract

It was the research group of Sheila Anne Bingham in Cambridge, United Kingdom, that showed for the first time that the consumption of red meat, in contrast to that of white meat, led to a strong increase in the fecal concentration of nitroso compounds in humans (Table 1) [9,10]. Furthermore, the above-mentioned research group demonstrated that the amount of nitroso compounds measured in the feces of the volunteers depended on the amount of red meat consumed, i.e., the higher the amount of red meat consumed, the higher were the concentrations of nitroso compounds measured in feces (Table 1) [9,10].

The indicated amount of meat was consumed by the volunteers (*n* = 8–17) each day for 10–15 days. In order to quantify the fecal concentration of the nitroso compounds, the total amount of feces excreted during the last 48 h was collected.

However; one should bear in mind that nitroso compounds are also formed when meat is strongly heated; so that the measured fecal concentrations represent the sum of the exogenous as well as the endogenous sources. If one considers that 600 g of fried red meat only contain about 13 µg of the nitroso compounds [11] and that much higher amounts of nitroso compounds were detected in feces of volunteers having consumed 600 g of red meat, one must conclude that most of the measured nitroso compounds were formed in the body of the volunteers.

Cross et al. [12] determined which constituent of red meat (heme or anorganic iron) stimulates the endogenous formation of nitroso compounds. Volunteers first received 60 g of red meat per day for 15 days, thereafter 60 g of red meat supplemented with 8 mg of heme per day for 15 days and in the end 60 g of red meat supplemented with 35 mg of ferrous gluconate per day for 15 days [12]. As shown in Table 2, the supplementation with heme led to a significant increase in the fecal concentration of nitroso compounds, while the supplementation with ferrous gluconate had no effect at all on the formation of nitroso compounds [12]. Furthermore, a complementary study showed that the consumption of a protein-rich vegetarian diet did not enhance the fecal concentration of nitroso compounds in humans [12]. Taken together, these results indicate that it is heme and not iron or protein that leads to an increase in the fecal concentration of nitroso compounds in humans. The important role of heme in the endogenous formation of nitroso compounds is supported by the observation that the consumption of white meat does not enhance the concentration of nitroso compounds in feces (Table 1), which is in turn explained by the fact that the concentration of heme in white meat is much lower than that in red meat (~20 nmol/g chicken meat compared to ~500 nmol/g beef steak) [13].

The volunteers consumed 60 g of red meat per day for 15 days, thereafter 60 g of red meat supplemented with 8 mg of heme per day for 15 days and in the end 60 g of red meat supplemented with 35 mg of ferrous gluconate per day for 15 days. Fecal samples, which had been collected on days 10, 13 and 15 were used for the quantification of fecal nitroso compounds.

Bacteria may also contribute to the formation of nitroso compounds in the gastrointestinal tract. Massey et al. [14] demonstrated about 20 years ago that in germ-free rats the endogenous formation of nitroso compounds only occurs in the presence of the normal intestinal microflora. Calmels et al. [15,16,17] showed that a number of bacterial strains, among others also some isolated from human feces samples, were able to catalyze the endogenous formation of nitroso compounds, depending on the strain, by making use of a nitrate or nitrite reductase at a neutral pH. However, this activity strongly varies among humans [18], and these variations could explain the great differences in the amount of nitroso compounds quantified in the feces samples of the different volunteers (note the large standard deviations in Table 1 and Table 2), although they had consumed the same amount of red meat.

The endogenous formation of nitroso compounds has also been demonstrated in feces samples of patients with ileostomy [19]. It should be pointed out that the average amount of nitroso compounds measured in the feces samples of the above-mentioned group of patients was very similar to that measured in the feces samples of healthy volunteers. These results suggest that nitroso compounds are not only formed by bacteria present in the large intestine [15] but are also formed by bacteria in the small intestine.

*N*-nitrosamines, *S*-nitrosothiols as well as the nitrosyl heme belong to the group of so-called endogenously formed nitroso compounds. In the early studies of Sheila A. Bingham’s research group, it was not possible to identify the endogenously formed nitroso compounds with the analytical methods available at that time. First in 2007, Kuhnle et al. [20] succeeded in showing that *S*-nitrosothiols and the nitrosyl heme are indeed formed in the human gastrointestinal tract and excreted via feces following the consumption of red meat.

Complementary in vitro experiments suggest that in a first step *S*-nitrosothiols are formed in the acid environment of the stomach [20]. As soon as the *S*-nitrosothiols reach the alkaline milieu of the small intestine, they become unstable and can be degraded, e.g., to nitric oxide via a copper (II)-catalyzed reaction [21]. Under alkaline conditions, nitric oxide may in part react with heme molecules [22], so that the nitrosyl heme is formed [20].

## 3. The Role of Endogenously Formed Nitroso Compounds in Colon Cancer Development

On the one hand, heme may take up nitric oxide; on the other hand, the nitrosyl heme may act, as in the case of *S*-nitrosothiols, as a nitric oxide donor [23,24]. It has been shown that nitric oxide derived from *S*-nitrosothiols and the nitrosyl heme by itself may support the proliferation and metastasis of tumor cells [25,26]. Moreover, it has also been documented that *S*-nitrosothiols and the nitrosyl heme as nitric oxide donors are able to induce the nitrosation of various molecules, e.g., the amino acid glycine [27]. In vitro experiments by Cupid et al. [27] have shown that in a subsequent reaction *N*-nitrosoglycine might give rise to the highly reactive alkylating agent diazoacetate, which in turn is converted to short-lived reaction products. These compounds are then able to bind to DNA bases, thereby forming stable DNA adducts [27,28]. Among the adducts having been detected are *O*^6^-carboxymethyl-2’-desoxyguanosine and *O*^6^-methyl-2’-desoxyguanosine [27].

In line with the above-mentioned in vitro observations, *O*^6^-carboxymethyl-2’-desoxyguanosine was unequivocally detected in DNA extracted from blood samples of three volunteers that had consumed 420 g of red meat per day for 43 days [27]. Moreover, in a further study [28] 21 volunteers consumed a vegetarian diet for 10 days and thereafter consumed 420 g of red meat per day for another 10 days. By making use of an immunocytochemical technique, Lewin et al. [28] were able to show that the number of *O*^6^-carboxymethylguanine-positive epithelial cells in feces strongly increased after the consumption of red meat. Furthermore, a positive correlation between the concentration of nitroso compounds and the percentage of *O*^6^-carboxymethylguanine-positive epithelial cells in feces was evident in the above-mentioned group of volunteers [28]. In a study by Le Leu et al. [29], twenty-three volunteers consumed 300 g of red meat per day over a four-week period. At the end of the four-week period, the consumption of a high red meat diet led to a statistically significant increase of the *O*^6^-methylguanine adduct levels in the rectal crypts when compared to its baseline by 21% [29].

The DNA repair enzyme *O*^6^-methylguanine-DNAmethyltransferase removes the *O*^6^-methylguanine adducts by transferring the methyl group from guanine to its active site, which leads to the inactivation and subsequent degradation of the enzyme [30,31]. Regarding the relevance of carboxymethylated DNA bases for colon cancer development, Shuker and Margison [32] reported that the *O*^6^-methylguanine-DNAmethyltransferase present in extracts of the human lung fibroblast cell line MRC-5 cannot repair carboxymethylated DNA bases and based on this finding hypothesized that carboxymethylated DNA bases may accumulate in different sections of the gastrointestinal tract after the consumption of high amounts of red meat. In contrast, Senthong et al. [33] showed that synthetic oligodeoxyribonucleotides containing *O*^6^-carboxymethylguanine effectively inactivate the *O*^6^-methylguanine-DNAmethyltransferase in a cell-free system and concluded that *O*^6^-carboxymethylguanine is an *O*^6^-methylguanine-DNAmethyltransferase substrate. Hence, whether the *O*^6^-methylguanine-DNAmethyltransferase is indeed able to repair the *O*^6^-carboxymethylguanine adducts in epithelial cells of the human colon and rectum remains unclear at the present time.

Gottschalg et al. [34] demonstrated that the incubation of a plasmid containing the human *p53* gene sequence with diazoacetate, which plays an important role in the formation of carboxymethylated DNA bases, led to a series of mutations in the *p53* gene, the observed *p53* gene mutation spectrum being almost identical to that in human colorectal tumors.

## 4. The Role of Lipid Peroxidation Products in Colon Cancer Development

More than ten years ago, Tappel [35] suggested that heme iron may catalyze the oxidative damage of lipids, thereby leading to so-called “oxidative chain reactions” and subsequently to the initiation of cancer in various different organs including the colon. Among the lipid peroxidation products thought to contribute to colorectal cancer development are the cytotoxic and genotoxic aldehydes malondialdehyde and 4-hydroxynonenal [36]. In a later study by Bastide et al. [37], the mechanisms by which red meat could contribute to the formation of colorectal tumors were analyzed. In a first step, these authors showed that a diet supplemented with 2.5% hemoglobin led to genotoxicity in the colon mucosa of *Apc^Min^*^/+^ mice and to an increased tumor load in these animals [37]. Furthermore, fecal water from rats given hemoglobin was rich in aldehydes and cytotoxic to wild-type *Apc*^+/+^ cells, but not to premalignant *Apc^Min^*^/+^ cells, while the aldehydes 4-hydroxynonenal and 4-hydroxyhexenal were more toxic to *Apc*^+/+^ cells than to *Apc^Min^*^/+^ cells and were only genotoxic to *Apc*^+/+^ cells [37]. Based on the results obtained, Bastide et al. [37] concluded that heme is the driving force in the red meat-mediated promotion of colorectal carcinogenesis and that lipid peroxidation products such as the alkenals malondialdehyde and 4-hydroxynonenal are prominent players in this process.

In line with the above-mentioned hypothesis, Guéraud et al. [38] reported that increased levels of malondialdehyde and the mercapturic acid of 1,4-dihydroxynonane, a 4-hydroxynonenal metabolite, were measured in the urine of rats fed a heme iron-rich diet when compared to control animals, while Van Hecke et al. [39] detected higher concentrations of malondialdehyde in the gastrointestinal contents and colonic tissues of rats fed a red beef diet than in those of rats fed a chicken meat diet. Surya et al. [40] showed that fecal water of hemoglobin- and beef-fed rats containing malondialdehyde and 4-hydroxynonenal induced apoptosis to a greater extent in *Apc*^+/+^ cells than in *Apc^Min^*^/+^ cells, which are considered to be preneoplastic, and was accompanied by a stronger Nrf2-dependent antioxidant response, known to be activated by aldehydes such as 4-hydroxynonenal, in *Apc^Min^*^/+^ cells than in *Apc*^+/+^ cells. The authors concluded that the stronger Nrf2-dependent antioxidant response could explain the tumor promoting effect of red meat in colorectal carcinogenesis, i.e., by inducing a positive selection of preneoplastic cells [40]. In rats fed a diet supplemented with hemin for 21 days, the lipid peroxidation levels in fecal water were significantly higher than those in fecal water of control rats [41]. These higher luminal lipid peroxidation levels were associated with higher mucosal inflammation markers as revealed by an increased colonic myeloperoxidase activity and an enhanced expression of interleukin-6 and transforming growth factor-β as well as a higher DNA damage in the colonic mucosa of hemin-fed rats when compared to control rats [41].

It has been suggested that polyphenols present, e.g., in red wine as well as in certain plant extracts could inhibit the tumor promoting effect of red meat in colon carcinogenesis by inhibiting lipid peroxidation [37]. In fact, Bastide et al. [42] showed that red wine and pomegranate extracts added to cured meat and given to rats treated with the colon carcinogen azoxymethane led to a reduced number of preneoplastic lesions in the colon of the rats. Moreover, Martin et al. [43] reported that an antioxidant marinade was able to reduce beef-mediated tumor promotion in the colon of azoxymethane-initiated rats as well as *Apc^Min^*^/+^ mice if compared to rodents being fed non-marinated beef.

Hemeryck et al. [44] determined the spectrum of DNA adducts in the colon of rats fed a meat-based diet to compare the possible genotoxic effects of red and white meat. The consumed meat type altered the DNA adductome: The levels of 22 different DNA adduct types significantly increased upon the consumption of beef (when compared to chicken), and in the case of red and processed meat alkylation- as well lipid peroxidation-related DNA adducts were identified [44]. In a further study, Hemeryck et al. analyzed the DNA adducts formed when red meat was incubated with human colonic microbiota [45]. By doing so, a number of alkylation- and lipid peroxidation-related DNA adduct types were again detected [45]. Overall, the DNA adductome analyses support the concept that nitroso compounds as well as lipid peroxidation products might lead to the formation of DNA adducts, which in turn could increase colorectal cancer risk.

## 5. Conclusions

The consumption of high amounts of red meat is accompanied by a significant formation of *N*-nitrosothiols and the nitrosyl heme as well as by the carboxymethylation of DNA in epithelial cells of the gastrointestinal tract. If one takes into account that relatively high amounts of *N*-nitrosothiols and the nitrosyl heme are formed and that carboxymethylated adducts might accumulate, it is tempting to suggest that nitroso compounds may play an important role in colorectal carcinogenesis. However, experimental evidence is accumulating that lipid peroxidation products might also contribute to the malignant transformation of epithelial cells in the human colon and rectum.

## Figures and Tables

**Table 1:** Effect of meat consumption on the concentration of nitroso compounds in feces (modified from [9,10]).

	Amount and Type of Daily Consumed Meat				
	0 g Meat	60 g Red Meat	240 g Red Meat	420–600 g Red Meat	420–600 g White Meat
Nitroso compounds in feces (ng/g)	444 ± 60 ^a^	347 ± 61 ^a^	1516 ± 414 ^b^	2104 ± 1524 ^b^	759 ± 528 ^a^
Nitroso compounds in feces (µg/day)	54 ± 7 ^a^	52 ± 11 ^a^	159 ± 33 ^b^	249 ± 167 ^b^	87 ± 55 ^a^

^a,b^ Means with different superscript letters are significantly different at *p* < 0.05.

**Table 2:** Effect of heme and iron (II) on the concentration of nitroso compounds in feces (modified from [12]).

	Amount of Daily Consumed Red Meat ± Supplements		
	60 g Red Meat	60 g Red Meat + Heme	60 g Red Meat + Iron (II)
Nitroso compounds in feces (ng/g)	766 ± 233 ^a^	1438 ± 345 ^b^	852 ± 393 ^a^
Nitroso compounds in feces (µg/day)	77 ± 9 ^a^	157 ± 23 ^b^	61 ± 10 ^a^

^a,b^ Means with different superscript letters are significantly different at *p* < 0.05.
